# Suppression of NLR-mediated plant immune detection by bacterial pathogens

**DOI:** 10.1093/jxb/erad246

**Published:** 2023-07-10

**Authors:** José S Rufián, Javier Rueda-Blanco, Carmen R Beuzón, Javier Ruiz-Albert

**Affiliations:** Instituto de Hortofruticultura Subtropical y Mediterránea ‘La Mayora’, Universidad de Málaga-Consejo Superior de Investigaciones Científicas (IHSM-UMA-CSIC), Depto. Biología Celular, Genética y Fisiología, Málaga, Spain; Instituto de Hortofruticultura Subtropical y Mediterránea ‘La Mayora’, Universidad de Málaga-Consejo Superior de Investigaciones Científicas (IHSM-UMA-CSIC), Depto. Biología Celular, Genética y Fisiología, Málaga, Spain; Instituto de Hortofruticultura Subtropical y Mediterránea ‘La Mayora’, Universidad de Málaga-Consejo Superior de Investigaciones Científicas (IHSM-UMA-CSIC), Depto. Biología Celular, Genética y Fisiología, Málaga, Spain; CSIC, Spain

**Keywords:** Bacterial effectors, defense evasion, defense suppression, effector-triggered immunity, NLR proteins, PAMP-triggered immunity, plant immunity, type III secretion system

## Abstract

The plant immune system is constituted of two functionally interdependent branches that provide the plant with an effective defense against microbial pathogens. They can be considered separate since one detects extracellular pathogen-associated molecular patterns by means of receptors on the plant surface, while the other detects pathogen-secreted virulence effectors via intracellular receptors. Plant defense depending on both branches can be effectively suppressed by host-adapted microbial pathogens. In this review we focus on bacterially driven suppression of the latter, known as effector-triggered immunity (ETI) and dependent on diverse NOD-like receptors (NLRs). We examine how some effectors secreted by pathogenic bacteria carrying type III secretion systems can be subject to specific NLR-mediated detection, which can be evaded by the action of additional co-secreted effectors (suppressors), implying that virulence depends on the coordinated action of the whole repertoire of effectors of any given bacterium and their complex epistatic interactions within the plant. We consider how ETI activation can be avoided by using suppressors to directly alter compromised co-secreted effectors, modify plant defense-associated proteins, or occasionally both. We also comment on the potential assembly within the plant cell of multi-protein complexes comprising both bacterial effectors and defense protein targets.

## Teaser

The plant immune system detects pathogen-injected virulence effectors using intracellular receptors (NLRs), triggering a defense response. Host-adapted bacteria suppress this response with additional effectors modifying defense proteins and co-secreted effectors.

## Introduction

Plants have evolved immune systems that effectively identify the presence of the different types of pathogens and activate the corresponding defense response through signal transduction pathways, mounting a multi-pronged reaction to avoid infection. Correspondingly, microbial pathogens have evolved multiple mechanisms for the evasion of plant immunity, as a crucial strategy for infection (Wang *et al.*, 2022).

**Fig. 1.**
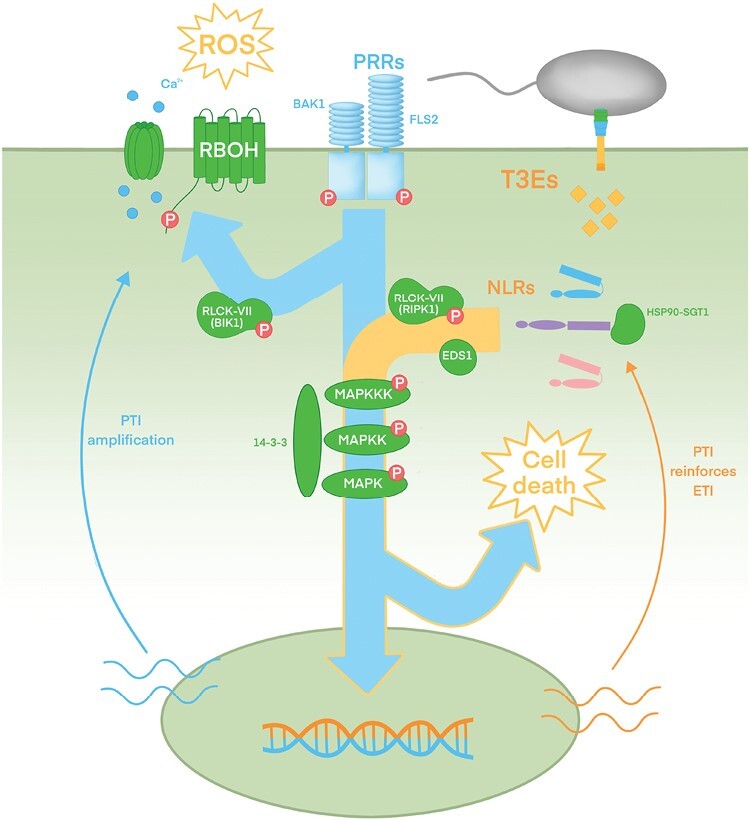
The plant defense system: interconnected ETI and PTI. Schematic view of PTI and ETI signaling components in the plant response against bacterial pathogens, and the interconnection between branches. Full arrows represent the defense signaling flow, with blue representing PRR-dependent signaling and orange representing NLR-dependent signaling. Stages common to both PTI and ETI are represented as an orange-trimmed blue arrow. Thin arrows represent the interconnection of PTI and ETI: right orange arrow indicates that PTI is required for a full NLR-dependent response (Ngou *et al.*, 2021; Yuan *et al.*, 2021), while left blue arrow indicates augmentation of PTI signal partially dependent on ETI components (Pruitt *et al.*, 2021; Tian *et al.*, 2021). PRRs are exemplified with the FLS2 receptor of bacterial flagellin and its co-receptor BAK1. Main components contributing to extracellular ROS signaling (RBOH and calcium channels) are depicted. RLCK-VII family of kinases is exemplified by BAK1 contributing to PTI and RIPK contributing to ETI. MAPKs cascades are represented generically, associated to scaffold proteins exemplified by the 14-3-3 protein family. MAPK modules potentially participate upstream of RLCKs (not depicted, see main text). NLRs are depicted in different colors to signify its diversity, and in either inactive (folded, denoting intramolecular interactions) or active (unfolded) states.

Plants cells can perceive pathogens in their immediate surroundings through membrane-spanning pattern-recognition receptors (PRRs) that detect extracellular microbe-derived elicitors, which usually are conserved, abundant microbial molecules collectively known as microbe- or pathogen-associated molecular patterns (MAMPs or PAMPs). The defense response mounted following PRR-dependent detection is commonly known as PAMP-triggered immunity (PTI). Many PRRs have been identified, each detecting a specific PAMP, allowing detection of many different microbial pathogens (DeFalco and Zipfel, 2021), with several different PRRs co-existing in the membrane of any given plant (Boutrot and Zipfel, 2017; Schellenberger *et al.* 2019). For example, bacterial pathogens are typically detected by PRRs such as FLS2 or EFR, which respectively recognize the 22-amino-acid peptide flg22 derived from bacterial flagellin or the elf-18 peptide derived from elongation factor 1 (Ef-Tu1) molecules acting as PAMPs (Boller and Felix, 2009). PRRs act in concert with membrane-bound co-receptors and other regulatory proteins. The signal perceived by PRRs is transduced downstream, mainly through phosphorylation events, to intracellular executors, among which receptor like cytoplasmic kinases (RLCKs) are paramount (DeFalco and Zipfel, 2021). In turn, RLCKs transduce the signal to other components downstream such as membrane-bound NADPH oxidases to induce reactive oxygen species (ROS) production, or MAP kinase modules to alter transcriptional programming of plant defense genes (Fig. 1).

Phytopathogenic bacteria possess several mechanisms that allow them to overcome the plant defense response. One of main bacterial virulence determinants is the type III secretion system (T3SS), a sort of molecular syringe that allows bacteria to inject proteins, generally known as type III effectors (T3Es), directly into the host cell, where they interfere with plant defense responses (Buttner, 2016). A survey of 181 host targets of bacterial T3Es suggested that as many as 80% of target proteins contribute to plant immunity (Khan *et al.*, 2018). Each bacterial strain possesses its own array of T3Es, which eventually determines the overall outcome of the infection process, depending on its interaction with the plant host genotype. To date, more than 50, 60, and 90 T3E families have been described, respectively, for the *Xanthomonas*, *Pseudomonas syringae*, and *Ralstonia solanacearum* species complexes, with some families being widespread among many bacterial strains, and some present only in a limited range of strains (Baltrus *et al.*, 2011; Peeters *et al.*, 2013; Dillon *et al.*, 2019; Timilsina *et al.*, 2020). The effector repertoire of each strain includes many T3Es that can cooperatively interfere with the signal transduction events following PRR-dependent recognition, thus suppressing PTI in susceptible plant genotypes.

Since bacterial effectors exert their functions within the host cell, pathogen perception can also occur intracellularly, where different T3Es can be detected by an array of plant resistance proteins (Adachi *et al.*, 2019; Jubic *et al.*, 2019). Most resistance proteins contain nucleotide-binding and leucine-rich repeat (NB-LRR) domains and are known as NB-LRR or NOD-like receptors (NLRs). Effector-triggered immunity (ETI), sometimes referred to as NLR-triggered immunity (NTI), is activated by direct or indirect pathogen effector recognition by NLRs, and there usually ensues a programmed cell death response (the hypersensitive response (HR)) at the site of infection, resulting in a drastic restriction of pathogen growth (Chiang and Coaker, 2015) (Fig. 1). Several models for NLR-mediated effector recognition have been assigned to individual NLRs, ranging from the direct recognition of the effector (direct model) to the recognition of the perturbation the effector exerts on its host interactor(s) (Kourelis and van der Hoorn, 2018). If the host protein modified by the T3E fulfills a function in the plant that constitutes a *bona fide* virulence target, then it is generically designated a ‘guardee’ (guard model). If the host protein modified by the T3E has similarity to the intended T3E target but fulfills no other purpose that activating defense when modified, it is defined as a decoy (decoy model). Since each plant genotype includes genes for a variable array of such NLR immune receptors, the outcome of any given infection depends on the complex network of epistatic interactions established between the specific secretome of the infecting bacterial strain and the specific NLR array of the host plant. Thus, a ‘compatible’ plant–bacteria interaction resulting in efficient pathogen multiplication might develop as a straightforward affair involving no recognition of T3Es due to the absence of suitable detecting NLRs in the plant genotype, or alternatively be the result of one or more events of NLR-dependent detection of T3Es concomitant with matching ETI-suppression by co-secreted effectors.

However, the classical depiction of plant immune signaling as two independent, separate pathways (PTI and ETI) has progressively been modified with the amassing of evidence of considerable interplay and synergy between PRR-dependent and NLR-dependent defense responses (Fig. 1). In Arabidopsis a fully-fledged NLR-dependent (ETI) response requires PRR-dependent activation (PTI) of downstream components such as membrane-bound NADPH oxidases or mitogen activated protein kinase (MAPK) modules (Ngou *et al.*, 2021; Yuan *et al.*, 2021). Additionally, a subset of PRRs establish a PTI defense response with the contribution of signaling components traditionally associated to ETI, such as the EDS1-PAD4 module (Adlung and Bonas, 2017; Pruitt *et al.*, 2021). This defense interplay has many implications for the analysis and characterization of plant–pathogen interactions and fits with the existence of several characterized T3Es that can suppress both PTI and ETI.

Here, we will review the ETI-suppressing activity of characterized T3Es from some archetypal bacterial phytopathogens, namely *Pseudomonas syringae*, *Xanthomonas*, and *Ralstonia*. We will discuss their respective host protein targets among the components of the plant defense-associated signal transduction pathways, but also T3Es’ biochemical modes of action, subcellular location, and interaction with co-secreted effectors, considering the potential assembly of complexes between co-secreted T3Es and plant-defense components. We will also consider the emerging characterization of T3E interaction at the secretome level via high-throughput approaches, and the implications for a crosstalk between co-secreted T3Es with or without ETI-suppressing activity. Finally, we will comment on the implications of the interplay between PRR-dependent and NLR-dependent defense responses on T3E-mediated defense suppression.

## First description of type III effector-dependent effector-triggered immunity suppression in a natural setting

Many T3Es have been described as displaying ETI-suppressing abilities, that is, they can suppress the ETI triggered by another T3E upon recognition by the corresponding NLR complex (Table 1). Since ETI usually ensures a programmed cell death response (HR) at the site of infection, T3SS-dependent triggering of HR by a bacterial strain or by independent expression of a single T3E has been extensively used as an indicator of ETI and effector-dependent suppression of HR as a method to identify ETI suppressors. While this is a valid approach, as extensively shown throughout this review, full ETI characterization should also include some form of disease resistance assay, and the use of cell death as the only proxy for ETI activation should be regarded with a degree of caution (Box 1). The methodology followed for the characterization of ETI and its corresponding suppression for each effector is listed in Table 1.

**Table 1.** Type III secretion system effectors with ETI-suppressing abilities

T3E	AKA	Bacterial strain	Similar ETI suppresors^*a*^	Suppresses ETI elicited by T3E^*b*^	Intra- secretome ^*c*^	Evidence of suppression^*d*^	Target linked to ETI suppression^*e*^	References
AvrBsT		*Xanthomonas*: *Xcv* 75-3	**YopJ** **family**	AvrBs1_Xcv85-10_	No	HR supp.	SnRK1	Szczesny *et al*. (2010)
AvrPphC	AvrB2	*P. syringae*: *Pph* 1449B		**AvrPphF**	Yes	Restores SD		Tsiamis *et al*. (2000)
AvrPphF	HopF1	*P. syringae*: *Pph* 1449B	**HopF** **family**	ND	Yes	HR supp. Restores SD Restores BG		Jackson *et al*. (1999) Tsiamis *et al*. (2000)
AvrRpt2		*P. syringae*: *Pto* T1		AvrRpm1_Pma_	No	Restores BG	AtRIN4	H. S. Kim *et al*. (2005), M. G. Kim *et al*. (2005)
HopAB1	AvrPtoB_B728a_	*P. syringae*: *Psy* B728A	**HopAB** **family**	HopAA1_PsyB728A_ HopAE1_PsyB728A_ AvrPto1 _PsyB728A_	Yes	HR supp.		Vinatzer *et al*. (2006),
HopAB2	AvrPtoB_DC3000_	*P. syringae*: *Pto* DC3000	**HopAB** **family**	HopA1_Pss61_	Yes	HR supp.		Jamir *et al*. (2004), Guo *et al*. (2009), Wei *et al*. (2018)
HopAF1		*P. syringae*: *Psy* B728A		**HopZ3**_**PsyB728A**_	Yes	HR supp.		Rufián *et al*. (2018a)
HopAM1	AvrPpiB1Pto	*P. syringae*: *Pto* DC3000		HopA1_Pss61_	No	HR supp.		Jamir *et al*. (2004), Guo *et al*. (2009)
HopAR1	AvrPphB	*P. syringae*: *Pph* 1302A		AvrB_Pgy_	No	Restores BG	AtRIPK GmRIPK	Russell *et al*. (2015)
HopBF1		*P. syringae*: *Psy* FF5		AvrRpm1_Pma_	No	HR supp.	HSP90	Hubert *et al*. (2003), Lopez *et al*. (2019)
HopD1	HopPtoD1	*P. syringae*: *Pto* DC3000	XopB HopD1	HopA1_Pss61_ AvrRpm1_Pma_ AvrRpt2_Pto-T1_ HopM1_PtoDC3000_ **HopAM1**_**PtoDC3000**_	No	HR supp.	AtNTL9	Jamir *et al*. (2004), Guo *et al*. (2009), Block *et al*. (2014), Wei *et al*. (2018)
HopE1	HopPtoE	*P. syringae*: *Pto* DC3000		HopA1_Pss61_ **HopAM1**_**PtoDC3000**_	No	HR supp.		Jamir *et al*. (2004), Guo *et al*. (2009), Wei *et al*. (2018)
HopF2	HopPtoF HopF1g	*P. syringae*: *Pto* DC3000	**HopF** **family**	HopA1_Pss61_ **AvrRpt2**_**Pto-T1**_ HopT1-2 _PtoDC3000_	Yes	HR supp. Restores SD Restores BG	RIN4	Jamir *et al*. (2004), Guo *et al*. (2009), Wilton *et al*. (2010), Wang *et al*. (2010), Martel *et al*. (2022)
HopG1	HopPtoG HopG1c	*P. syringae*: *Pto* DC3000		HopA1_Pss61_ HopT1-2_PtoDC3000_ HopM1_PtoDC3000_	Yes	HR supp. Restores SD Restores BG		Guo *et al*. (2009), Martel *et al*. (2022)
HopI1		*P. syringae*: *Pto* DC3000		AvrE1_PtoDC3000_ HopM1_PtoDC3000_ **HopQ1-1**_**PtoDC3000**_ HopR1_PtoDC3000_ **HopAM1**_**PtoDC3000**_	Yes	HR supp. Restores BG		Wei *et al*. (2018)
HopPtoK	HopK	*P. syringae*: *Pto* DC3000		HopA1_Pss61_ HopM1_PtoDC3000_	No	HR supp.		Jamir *et al*. (2004), Guo *et al*. (2009), Wei *et al*. (2018)
HopQ1	HopQ1-1 HopQ1a	*P. syringae*: *Pto* DC3000, *Pma* 4326	XopQ HopQ1	AvrPto1_Pcd9625_	No	Restores SD Restores BG		Martel *et al*. (2022)
HopS2		*P. syringae*: *Pto* DC3000		HopA1_Pss61_	No	HR supp.		Guo *et al*. (2009)
HopX1	AvrPphEPto	*P. syringae*: *Pto* DC3000		HopA1_Pss61_	No	HR supp.		Jamir *et al*. (2004), Guo *et al*. (2009)
HopZ1a		*P. syringae*: *Psy* 7B40	**YopJ** **family**	**AvrRpt2**_**Pto-T1**_ AvrRps4_Pph1448A_ AvrRpm1_Pma_	No	Restores BG	AtMKK7	Macho *et al*. (2010), Rufián *et al*. (2015), Rufián *et al*. (2021)
HopZ3		*P. syringae*: *Psy* B728A	**YopJ** **family**	AvrPto1_Psy_ HopAA1_Psy_ HopM1_Psy_ HopAE1_Psy_ AvrB3_Psy_ AvrRpm1_Psy_	Yes	HR supp.	AtRIN4, SlRIN4 AtRIPK, SlRIPK PTO FEN **AvrB3-Psy** **AvrRpm1-Psy** **AvrPto1-Psy**	Vinatzer *et al*. (2006), Lee *et al*. (2015), Jelenska *et al*. (2021)
RipAC	PopC	*Ralstonia* RS1000, GMI1000		RipAA_RS1000_ RipP1_RS1000_ RipE1_GMI1000_	Yes	HR supp. Restores BG	NbSGT1	Yu *et al*. (2020, 2022), Nakano *et al*. (2021)
RipAP		*Ralstonia* RS1000		RipAA_RS1000_	Yes	HR supp.		Nakano *et al*. (2021)
RipAU		*Ralstonia* RS1000		RipAA_RS1000_	Yes	HR supp.		Nakano *et al*. (2021)
RipI		*Ralstonia* RS1000		RipAA_RS1000_	Yes	HR supp.		Nakano *et al*. (2021)
VirPphA	HopAB1_Pph1449_	*P. syringae*: *Pph* 1449B	**HopAB** **family**	ND	Yes	HR supp. Restores SD Restores BG		Jackson *et al*. (1999), Tsiamis *et al*. (2000)
XopAC	AvrAC	*Xanthomonas*: *Xcc*		AvrB_Pgy_	No	HR supp. Restores BG	AtRIPK	Feng *et al*. (2012)
XopB		*Xanthomonas*: *Xcv* 85-10	XopB HopD1	**AvrBsT**_**Xcv75-3**_	No	HR supp. Restores BG		Schulze *et al*. (2012)
XopQ		*Xanthomonas*: *Xcv* 85-10, *Xoo*	XopQ HopQ1	ND	Yes	HR supp.	SlTFT4	Sinha *et al*. (2013), Teper *et al.* (2014)

^*a*^ Individual names are not stated when all similar T3Es can be assembled into a well-described family (bold).

^*b*^ ETI-eliciting T3Es that function themselves as ETI suppressors are highlighted in bold.

^*c*^ Yes: ETI-suppressing and ETI-eliciting T3Es belong to the secretome of the same strain, as reported in at least one of the references.

^*d*^ HR supp., suppression of HR; Restores SD, restoration of symptom development; Restores BG, restoration of bacterial growth.

^*e*^ Targets that correspond to co-secreted T3Es are highlighted in bold.

Box 1. **Technical considerations for effector-triggered immunity-suppression assays**

Several methodological caveats should be considered when analysing ETI suppression by T3Es. (i) Regarding the use of macroscopic cell death symptoms (macroscopic HR) for the characterization of ETI suppressors, it is important to notice that in Arabidopsis and other plant species HR, or at least HR-like macroscopic symptoms, might occasionally result from PAMP or MAMP recognition by PRRs (Bjornson and Zipfel, 2021); thus it should be confirmed that the detected macroscopic HR is directly dependent on the presence of a functional T3SS in the strain delivering the T3Es under study. (ii) When resorting to transgenic expression of T3Es in Arabidopsis or *Agrobacterium*-mediated transient expression in *Nicotiana*, it should be noted that non-physiological levels of T3E expression and/or interference between *Agrobacterium* cultures co-expressing both effectors might also influence the output, leading to variable results. (iii) Since macroscopic cell death symptoms are sometimes uncoupled from pathogen growth inhibition (Yu *et al.*, 1998; Greenberg *et al.*, 2000; Balague *et al.*, 2003; Jurkowski *et al.*, 2004; Coll *et al.*, 2010; Menna *et al.*, 2015; Lapin *et al.*, 2019; Martel *et al.*, 2022), effective suppression of immune activation should be confirmed by monitoring changes in bacterial growth, rather than exclusively relying on the presence or absence of macroscopic HR. (iv) When monitoring changes in bacterial growth to assay immune suppression, the dose of bacterial inoculation should also be taken into consideration, since it might alter the outcome of the ETI suppression analysis (Lee *et al.*, 2015).

The first described ETI-suppressing T3Es were VirPphA and AvrPphF (currently known as HopAB1_Pph_ and HopF1_Pph_, respectively), both present in a native plasmid of *Pseudomonas syringae* pathovar (pv) *phaseolicola* (*Pph*) strain 1449B (Jackson *et al.*, 1999; Tsiamis *et al.*, 2000). A plasmid cured *Pph* 1449B strain lost virulence towards previously susceptible bean cultivars, triggering an HR that, crucially, was dependent on the T3SS present in the genome, and therefore linked to ETI. Complementation with plasmid-encoded VirPphA_Pph1449B_ (Jackson *et al.*, 1999) or AvrPphF_Pph1449B_ (Tsiamis *et al.*, 2000) resulted in the suppression of HR and restoration of virulence. Suppression by each of these effectors was lost after knockout effector mutation (Jackson *et al.*, 1999; Tsiamis *et al.*, 2000). In other bean cultivars, with different genetic backgrounds, these ETI-suppressing effectors triggered ETI themselves, being detected by the corresponding plant resistance genes. This experimental model allowed for the demonstration of an additional feature of ETI suppression: a third effector encoded in the same native plasmid, AvrPphC_Pph1449B_ (also known as AvrB2_Pph_), was able to suppress the HR triggered by AvrPphF_Pph1449B_ in resistant cultivars (Tsiamis *et al.*, 2000), thus proving that an ETI suppressor can itself be ‘protected’ by another co-secreted effector (discussed below). A corollary from these seminal papers is that suppression of ETI is rather specific: ETI suppressing activities of AvrPphF_Pph1449B_ in a given cultivar does not prevent its recognition in another resistant cultivar (Tsiamis *et al.*, 2000).

## Screenings for effector-triggered immunity suppressors using expression of heterologous type III effectors

This early description of ETI suppression among co-secreted effectors belonging to the same bacterial T3SS secretome (intra-secretome or within-strain suppression) was followed by several screenings analysing the suppression of ETI triggered by heterologous effectors. In this kind of experimental approach, the T3E used to trigger ETI and the suppressor T3E being assayed belong to different bacterial strains, and thus are not normally co-secreted from the same strain. Such heterologous screenings were preceded by a report of the ability of heterologously expressed *P. syringae* T3E AvrRpt2 to be epistatic over the HR triggered by another T3E, AvrRpm1 (Reuber and Ausubel, 1996; Ritter and Dangl, 1996). This kind of approach, while allowing for the identification of novel ETI-suppressing effectors and facilitating the characterization of the molecular mechanisms involved in suppression and the plant targets being interfered, should be regarded with a degree of caution.

Jamir *et al.* (2004) used a non-pathogenic *Pseudomonas fluorescens* carrying the cosmid pHIR11, containing a 25 kb region of *P. syringae* pv. *syringae* strain 61 (*Pss* 61) encoding a pathogenicity island expressing a functional T3SS and the effector HopA1_Pss61_. The *P. fluorescens* (pHIR11) strain translocates the effector, thus inducing HopA1_Pss61_-triggered HR in tobacco plants. Using this experimental setting, 19 effectors of *P. syringae* pv. *tomato* strain DC3000 (*Pto* DC3000) were expressed, screening their individual potential to suppress HopA1_Pss61_-induced HR. Five *Pto* DC3000 effectors, namely AvrPphE_Pto_, AvrPpiB1_Pto_, HopPtoE, AvrPtoB, and HopPtoF (currently HopX1, HopAM1-1, HopE1, HopAB2, and HopF2) could each completely suppress HopA1_Pss61_-induced ETI, while two additional effectors, HopPtoD1 and HopPtoK (currently HopD1 and HopK1), achieved partial suppression (Jamir *et al.*, 2004). Later, a similar approach by Guo *et al.* (2009) confirmed the ability of these seven previously identified *Pto* effectors to suppress HopA1_Pss61_-triggered HR and added effector HopS2_PtoDC3000_ to the list. Interestingly, HopF2_PtoDC3000_ and AvrPtoB_PtoDC3000_ (Jamir *et al.*, 2004) belong to the same effector families as AvrPphF_Pph1448B_ and VirPphA_Pph1448B_, respectively. This work proposed further candidates achieving partial suppression when using lesser inoculum doses of *P. fluorescens* (pHIR11) that might be regarded as ETI suppressors with lower confidence pending further analysis (Guo *et al.*, 2009). This is the case of HopPtoD1 (currently HopD1_PtoDC3000_), which was later shown to support increased growth of *P. syringae* strains triggering AvrRpm1- and AvrRpt2-dependent ETI (Block *et al.*, 2014).

In a similar fashion, a recent high throughput screening (Laflamme *et al.*, 2020) defined a set (designated PsyTEC) of 529 T3E effectors from different *P. syringae* strains, representative of the 4636 protein sequences available to date. Each of these T3Es was ectopically expressed from *Pto* DC3000 bacteria inoculated into Arabidopsis, leading to the identification of 59 effectors that trigger ETI in the model plant. Interestingly, when each of these 59 effectors were delivered into the same host plant but from a different strain, *P. syringae* pv. *maculicola* ES4326 (*Pma* ES4326), several failed to trigger immunity, suggesting that *Pma* ES4326 encodes effectors capable of suppressing these ETIs, which are encoded by *Pto* DC3000. In a follow-up work (Martel *et al.*, 2022) the reciprocal experiment was performed: the PsyTEC set was expressed from *Pma* ES4326 bacteria and tested in Arabidopsis, resulting in the identification of 60 effectors that triggered ETI. When each of these 60 effectors was delivered from *Pto* DC3000, 10 (including effectors belonging to the AvrPto1 and HopT1 families) failed to induce immunity, supporting the notion that *Pto* DC3000 in turn encodes effector(s) capable of suppressing these ETIs, which are thus not encoded by *Pma* ES4326. Co-expression of either AvrPto1 or HopT1 with each of the 29 effectors encoded by *Pto* DC3000 in *Pma* ES4326 revealed ETI-suppression activity for two additional ETI-suppressing *Pto* DC3000 effectors, namely HopQ1_PtoDC3000_ and HopF_PtoDC3000_, respectively, and confirmed previous reports on HopG1_PtoDC3000_ activity as an ETI suppressor (Guo *et al.*, 2009; Martel *et al.*, 2022). By analysing ETI suppression between heterologous effectors (i.e. effectors not naturally encoded by the same strain), these high-throughput experiments confirm that elicitation of ETI by expression of a given T3E depends on the repertoire of effectors of the delivering strain, likely due to the presence or absence of specific ETI-suppressing effectors, and not only on the repertoire of NLR genes of the inoculated plant genotype.

## Intra-secretome suppression in natural pathosystems

While heterologous expression in model pathosystems might provide valuable information regarding ETI suppression, experimental approaches involving T3Es encoded by the same strain, preferably in the context of natural pathosystems, contribute to ascertaining the biological context, relevance, and complexity of ETI suppression. Since intra-secretome (within-strain) suppression more accurately reflects the biological relevance of ETI suppression in a natural environment, we specify as a subscript the strain of origin when needed.

Several effectors belonging to the secretome of *P. syringae* pv. *syringae* B728a (*Psy* B728a), including HopAA1, HopM1, HopAE1, and AvrPto1, trigger ETI when transiently expressed in *Nicotiana benthamiana* and/or bean (*Phaseolus vulgaris)*, both considered host plants for *Psy* B728a (Vinatzer *et al.*, 2006). Transiently expressed HopAA1_B728a_ induces cell death in *N. benthamiana*, and the corresponding *Psy* B728a deletion mutant shows enhanced growth in the same plant, suggesting that HopAA1-induced ETI limits bacterial growth in this compatible host and is therefore not completely suppressed by other effectors of *Psy* B728a (Vinatzer *et al.*, 2006). In contrast, although transient expression of HopM1_B728a_ also induces cell death in *N. benthamiana*, deletion of *hopM1* does not have any impact on *Psy* B728a virulence, suggesting that HopM1-triggered ETI is fully suppressed by other co-secreted effector(s) encoded by *Psy* B728a (Vinatzer *et al.*, 2006). A screening based on transient co-expression in *N. benthamiana* of each of the effectors from *Psy* B728a that trigger ETI when transiently expressed in this host with each of the remaining effectors of this strain revealed that HopAB1 suppresses cell death triggered by HopAA1, HopAE1, and AvrPto1, while HopZ3 also suppresses cell death induced by HopM1 (Vinatzer *et al.*, 2006).

Interestingly, when the same set of effectors is analysed in a different *Psy* B728a plant host, additional effector–host plant interplay is revealed. For example, HopZ3_PsyB728a_ triggers ETI when expressed in bean (Rufián *et al.*, 2018a; Vinatzer *et al.*, 2006). However, *hopZ3* deletion does not affect *Psy* B728a growth in bean, suggesting that HopZ3-triggered ETI can be suppressed by co-secreted effector(s) of the *Psy* B728a secretome. This is supported by the fact that when HopZ3_PsyB728a_ is expressed from the efficient bean pathogen *P. syringae* pv. *phaseolicola* strain 1448A (*Pph* 1448A), which does not naturally encode this effector, it causes a decrease in bacterial growth and symptom development in bean, fitting with HopZ3-triggered immunity in bean not being suppressed by any of the effectors encoded by *Pph* 1448A (Rufián *et al.*, 2018a). A screening based on transient co-expression in bean of HopZ3_PsyB728a_ with each effector of the *Psy* B728a secretome revealed that HopZ3-triggered cell death is partially suppressed by at least five *Psy* B728a co-secreted effectors, with HopAF1 achieving the strongest individual suppression (Rufián *et al.*, 2018a).

Another intra-secretome approach, in this case taking advantage of the ability of *Pto* DC3000 to efficiently infect Arabidopsis, backed by co-expression in *N. benthamiana*, revealed that HopI1_PtoDC3000_ suppresses ETI triggered by at least five effectors of the same secretome, namely AvrE1, HopM1, HopQ1-1, HopR1, and HopAM1, and confirmed AvrPtoB (HopAB2) as an ETI suppressor (Jamir *et al.*, 2004; Wei *et al.*, 2018). Such an intra-secretome approach in a natural context is not always feasible. For instance, the *P. syringae* ETI suppressor HopZ1a (see below) has been exhaustively characterized almost exclusively by heterologous expression in model pathosystems, likely because the bacterial strains natively carrying HopZ1a are poorly characterized and/or have been isolated from technically challenging host plants (Ma *et al.*, 2006).

Only a few of the ETI suppressors have been characterized beyond the description of their ability to suppress the HR and/or bacterial growth limitation elicited by another effector in one or more hosts and/or delivery systems. In the next sections we will describe the targets and molecular mechanisms used by these characterized effectors to interfere with the plant immune system.

## Suppression of effector-triggered immunity by targeting chaperone complexes

Components of the NLR-associated immune signaling complexes, responsible for immune recognition, are obvious targets for ETI-suppressing T3Es. Interestingly ETI suppressors do not seem to interfere directly with NLRs, but rather with associated defense components, such as molecular chaperones, decoys, or ‘guardee’ proteins.

NLR proteins are in a latent, inactive state through both intra-molecular interactions between their own NB and LRR domains, and inter-molecular interactions with chaperone complexes. Some T3Es target molecular chaperones to suppress ETI. NLRs become activated after direct detection of the T3E, or detection of T3E activity on the corresponding target or decoy, to avoid a constitutively active immune response, which has a negative effect on plant fitness. The RAR1–SGT1–HSP90 chaperone complex contributes to NLR-triggered immunity, seemingly by facilitating the assembly of NLR activation complexes, with knockout or silencing of its individual components compromising resistance against several pathogens (Kadota and Shirasu, 2012) (Fig. 1).

**Fig. 2.**
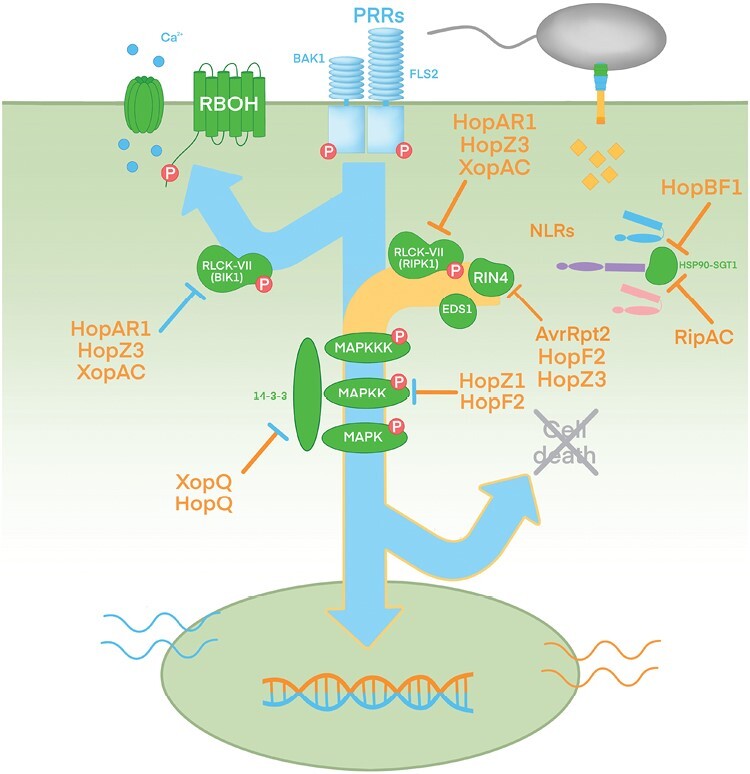
T3Es acting as ETI suppressors on known plant target proteins. Type III secreted bacterial effectors (T3Es) with characterized plant targets are depicted in a schematic view of the plant defense response. Suppression of ETI-associated targets (RIPK, RIN4, HSP90, SGT1) is represented with orange bars, suppression of PTI-associated targets (BIK1) is represented with blue bars, and suppression of targets common to both branches (MAPK modules and scaffolds) is indicated with blue-tipped orange bars. Some effectors (HopAR1, HopZ3, XopAC) are depicted more than once since they interference with several plant targets.

*Pseudomonas syringae* effector HopBF1_PsyFF5_ phosphorylates HSP90 to suppress NLR activation (Lopez *et al.*, 2019) (Fig. 2). HopBF1 adopts a minimal protein kinase fold that is recognized by HSP90 as a host client protein, then phosphorylates HSP90 inhibiting its ATPase activity, thus rendering the chaperone almost completely inactive (Lopez *et al.*, 2019). The specific residue of HSP90 that is modified by HopBF1_PsyFF5_ had been previously identified in a genetic screen for Arabidopsis mutants impaired in the immunity response triggered by AvrRpm1_Pma_ and mediated by the NLR RESISTANCE TO P. SYRINGAE PV MACULICOLA 1 (RPM1) (Hubert *et al.*, 2003). HopBF1_PsyFF5_, but not its catalytically inactive version, is able to suppress the widespread hypersensitive response induced *in planta* by expression of an auto-active mutant of RPM1 (Gao *et al.*, 2011) during co-expression in *N. benthamiana* (Lopez *et al.*, 2019). Since both agroinfiltration and natural delivery of HopBF1_PsyFF5_ during infection cause widespread tissue collapse and necrosis in *Nicotiana* and Arabidopsis plants, while HopBF1_PsyFF5_ suppresses AvrRpm1-triggered immunity it also seems to induce host cell death itself during the late stage of infection. It remains to be investigated whether this late, HopBF1_PsyFF5_-induced host cell death can be suppressed when HopBF1 is co-secreted with the rest of the *Psy* FF5 effector repertoire in the context of natural infection. It will also be worth testing whether HopBF1 can interfere with the assembly of immune complexes other than RPM1, considering that HSP90 chaperone activity is not exclusive on RPM1, and if so, the identities of the T3Es triggering the corresponding ETIs.

*Ralstonia* effector RipAC (formerly PopC) targets SGT1 to suppress RipAA_RS1000_-, RipP1_RS1000_-, and RipE1_GMI1000_-triggered ETI in *N. benthamiana* (Yu *et al*., 2020; Nakano *et al*., 2021) (Fig. 2). RipAC was previously identified as one of 11 *Ralstonia* RS1000 effectors that can suppress PTI responses (Nakano and Mukaihara, 2019), which were later tested by transient expression in *N. benthamiana* for their ability to suppress RipAA_RS1000_-triggered ETI. Out of the 11 effectors tested, four were able to suppress RipAA-triggered ETI, including RipAC but also RipI, RipAP, and RipAU. RipAC contains tandem repeats of a LRR domain that is essential for its interaction with NbSGT1 and the subsequent ETI suppression (Nakano *et al*., 2021). RipAC markedly inhibits the interaction between NbSGT1 and NbRAR1, in a manner dependent on the LRR domain of RipAC (Nakano *et al*., 2021). RipAA- and RipP1-triggered ETI is dependent on the presence of NbSGT1 since it is not observed in NbSGT1-silenced plants (Nakano *et al*., 2021). RipAC_GMI1000_ also suppresses RipE1_GMI1000_-induced HR by transient co-expression in *N. benthamiana*, likely by interfering with MAPK-mediated phosphorylation of SGT1, which is required for RipE1-induced HR (Sang *et al.*, 2020; Yu *et al*., 2020). Interestingly, SGT1 is not required for PTI responses in *N. benthamiana* and Arabidopsis (Nakano *et al*., 2021; Yu *et al*., 2021), and thus RipAC is likely to target additional host factor(s) to suppress PTI.

While identified during the same screening, the ETI-suppressing abilities of RipI_RS1000_, RipAP_RS1000_, and RipAU_RS1000_ have not been characterized further. Recently, RipI_GM1000_ was described to interact with glutamate decarboxylases to alter plant metabolism and support bacterial growth (Xian *et al.*, 2020). This highlights the fact that many T3Es target multiple host proteins, sometimes interfering with very different host pathways to the benefit of the pathogen (discussed below).

## Decoys, ‘guardees’, and NLR-associated kinases as targets for effector-triggered immunity suppression

Other ETI suppressors target components of the plant defense system responsible for signal integration, common to PTI and ETI, such as RLCKs or MAPK cascades (see below). Interestingly, it has been estimated that kinases might account for approximately 30% of T3E plant targets (Khan *et al.*, 2018).

**Fig. 3.**
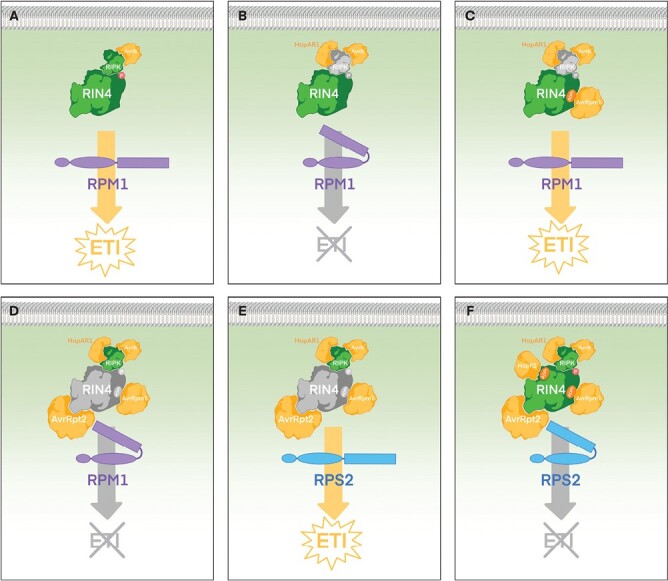
Multifaceted interplay among T3Es and the plant immune system: the RIN4 immune complex example (AvrB, AvrRpm1, AvrRpt2, and HopF2). The outcome of the infection process depends on the interaction amongst the T3E repertoire of the bacterial pathogen and the NLR array encoded by the plant host genotype. This is illustrated here by the different potential outputs of the interaction between the RIN4-associated immune complex and effectors AvrB, AvrPm1, AvrRpt2, and HopF2. Only a few of the potential combinations of T3Es and NLRs are represented, even for the limited number of participants included in this example. The interplay grows increasingly complex as the numbers of T3Es and NLRs multiply. Illustrations of T3Es and plant targets are not meant to depict actual protein structures. NLRs are depicted as folded when inactive (denoting intramolecular interactions). (A) Secreted T3E AvrB induces RIPK-dependent phosphorylation (P in red) of RIN4, which is detected in plant genotypes encoding the NLR RPM1, triggering ETI. (B) Co-secreted HopAR1 degrades RIPK (grey), suppressing AvrB-dependent ETI. (C) Secreted AvrRpm1 ADP-ribosylates (ADPr in orange) RIN4, which is detected by RPM1 triggering ETI, even in the presence of co-secreted HopAR1 and/or AvrB. (D) Co-secreted AvrRpt2 degrades RIN4 (grey), suppressing AvrB- and AvrRpm1-dependent ETI. (E) In plant genotypes encoding the NLR RPS2, AvrRpt2-dependent degradation of RIN4 is detected by RPS2, triggering ETI. (F) Co-secreted HopF2 ADP-ribosylates RIN4, avoiding AvrRpt2-dependent degradation of RIN4, thus suppressing AvrRpt2-dependent ETI in RPS2 plant genotypes.

Some ETI suppressors target signaling components of NLR immune complexes. This has been well characterized for several T3Es targeting the RPM1 immune complex. This complex comprises the NLR RPM1 acting in concert with the RLCK RPM1-INTERACTING PROTEIN KINASE (RIPK), together guarding the sensor protein RIN4. RIN4 could be considered an immune hub since it is affected directly or indirectly by many different T3Es, regulating the defense response acting as a phospho-switch platform (Mackey *et al.*, 2002, 2003; Wilton *et al.*, 2010; Liu *et al.*, 2011; Chung *et al.*, 2014; Choi *et al.*, 2021). *Pseudomonas syringae* T3Es AvrRpm1_Pma_ and AvrRpt2_PtoT1_ suppress PTI and enhance bacterial growth in susceptible plants lacking the NLRs RPM1 and RPS2, respectively (M. G. Kim *et al.*, 2005). AvrRpm1_Pma_ ADP-ribosylates RIN4 and induces its phosphorylation (Mackey *et al.*, 2002; Redditt *et al.*, 2019). However, in plant genotypes expressing RPM1, AvrRpm1_Pma_-dependent stimulation of RIN4 phosphorylation results in the induction of RPM1-dependent ETI (Mackey *et al.*, 2002) (Fig. 3). *Pseudomonas syringae* T3E AvrRpt2_PtoT1_ functions as an ETI suppressor of AvrRpm1_Pma_-triggered RPM1-dependent defense response, by cleaving RIN4 with its cysteine protease activity (Coaker *et al.*, 2005; H. S. Kim *et al.*, 2005) (Fig. 3).

*Pseudomonas syringae* T3E AvrB_Pgy_ also triggers RPM1-dependent ETI, since AvrB_Pgy_ association with RIPK stimulates RIPK-dependent phosphorylation of RIN4 (Liu *et al.*, 2011) (Fig. 3). *Pseudomonas syringae* effector HopAR1_Pph_ (also known as AvrPphB) suppresses AvrB_Pgy_-triggered ETI (Russell *et al.*, 2015). HopAR1_Pph_ is a cysteine protease that cleaves RIPK in a conserved motif present in other RLCKs, thus blocking AvrB_Pgy_-induced RIPK-dependent phosphorylation of RIN4, and therefore suppressing AvrB_Pgy_ (but not AvrRpm1_Pma_)-triggered RPM1-dependent immunity (Russell *et al.*, 2015) (Fig. 3). HopAR1_Pph_ failure to suppress AvrRpm1_Pma_-triggered ETI, suggests that RIPK is not necessary for AvrRpm1_Pma_-dependent phosphorylation of RIN4 (Russell *et al.*, 2015). HopAR1_Pph_ can also suppress the ETI triggered by AvrB_Pgy_ in resistant soybean cultivars expressing the resistance protein Rpg1b, suggesting that soybean employs a RIPK homolog in the Rpg1b-meditated detection of AvrB_Pgy_ (Ashfield *et al.*, 1995; Russell *et al.*, 2015). Nonetheless, soybean RIN4 homologs are involved in Rpg1b-dependent signaling (Russell *et al.*, 2015). Interestingly, the ETI-eliciting AvrB_Pgy_ belongs to the same T3E family as ETI suppressor AvrPphC_Pph_ (AvrB2_Pph_) discussed above. It is appealing to speculate that AvrPphC_Pph_ might be targeting a bean homologue of RIPK to achieve ETI suppression.

**Fig. 4.**
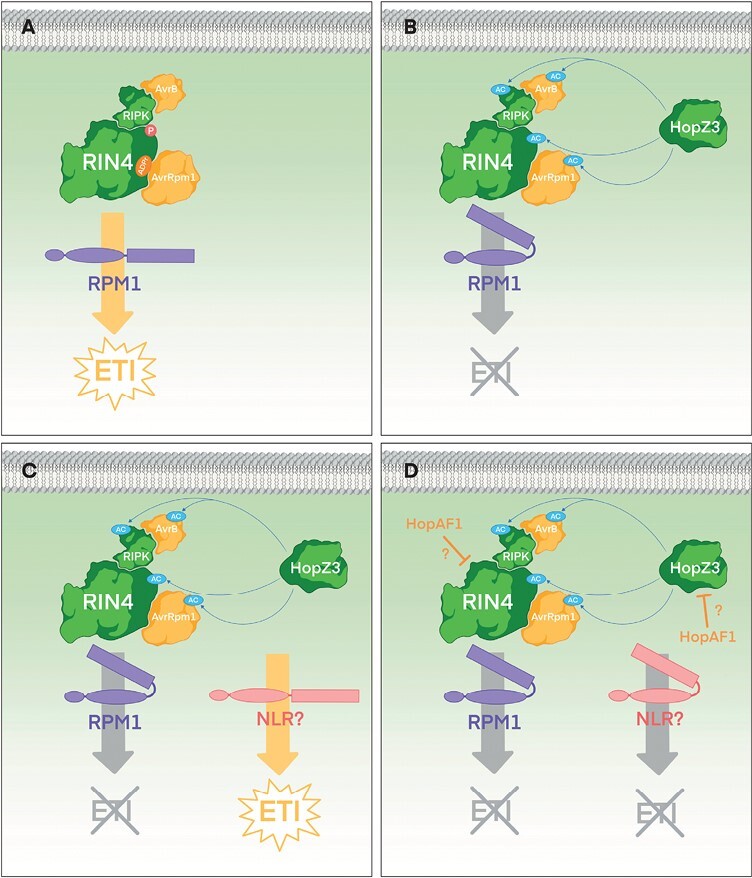
Multifaceted interplay among T3Es and the plant immune system: the RIN4 immune complex example (AvrB, AvrRpm1, HopZ3, and HopAF1). The figure illustrates another example of the interplay amongst bacterial T3Es and plant NLRs, also in the context of the RIN4 immunity complex. Illustrations of T3Es and plant targets are not meant to depict actual protein structures. NLRs are depicted as folded when inactive (denoting intramolecular interactions). (A) Co-secreted T3Es AvrB and AvrRpm1 induce alterations of RIN4, in an RIPK-dependent and -independent manner, respectively, which are detected in plant genotypes encoding the NLR RPM1, triggering ETI. (B) When co-secreted, HopZ3 acetylates (AC in pale blue) RIPK, RIN4, AvrB, and AvrRpm1, suppressing ETI. (C) In plant genotypes encoding a still undescribed NLR, HopZ3 interference with the complex is detected, triggering HopZ3-dependent ETI. (D) Co-secreted HopAF1 suppresses HopZ3-dependent ETI, but the molecular mechanism is still uncharacterized.

Unlike more specific AvrRpt2 or HopAR1 suppression of RPM1-mediated ETI, *P. syringae* effector HopZ3_PsyB728a_ can suppress both AvrRpm1_Psy_- and AvrB3_Psy_-triggered RPM1-mediated immunity (Lee *et al.*, 2015). HopZ3_PsyB728a_ interacts with and acetylates both RIN4 and RIPK (Lee *et al.*, 2015) (Fig. 4). HopZ3_PsyB728a_ acetylation of key residues in RIPK inhibit its kinase activity, while acetylation of RIN4 reduces the susceptibility of RIN4 to be phosphorylated by RIPK (Lee *et al.*, 2015). Thus, HopZ3_PsyB728a_ suppresses RIN4 phosphorylation triggered by AvrRpm1_Psy_ and AvrB3_Psy_ that leads to RPM1 immune activation (Fig. 4). Interestingly, while HopZ3_PsyB728a_ and HopAR1_Pph_ modes of action differ, both share RIPK as target protein, and both achieve the same effect, which is reducing RIN4 phosphorylation and thus suppression of RPM1-mediated ETI. *Xanthomonas* effector XopAC_Xcc_ (previously known as AvrAC_Xcc_) can also suppress RPM1-dependent ETI by interfering with RIPK, in this case by uridylating the same key residues that *P. syringae* HopZ3_PsyB728a_ modifies by acetylation (Feng *et al*., 2012; Lee *et al.*, 2015). This makes a third T3E targeting the same plant protein to suppress RPM1-dependent ETI, belonging to a different T3E family, with three different modes of action, and in the case of XopAC_Xcc_ belonging to a different bacterial species. It is important to notice that the ETI-suppressing phenotype of XopAC_Xcc_ was analysed using AvrB_Pgy_ from *P. syringae* as ETI-triggering effector (Feng *et al*., 2012), and thus it remains to be demonstrated if XopAC_Xcc_ contributes to suppressing ETI triggered by another T3E from the same secretome in *Xanthomonas*.

While the abovementioned data were obtained in Arabidopsis, interference with an analogous decoy-associated immune complex has been described also in tomato. Tomato plants lack RPM1 but contain the NLR protein PSEUDOMONAS RESISTANCE AND FENTHION SENSITIVITY (PRF), forming an immune complex with PSEUDOMONAS SYRINGAE PV TOMATO RESISTANCE (PTO) and FENTHION SENSITIVITY (FEN), two cytoplasmic protein kinases of the RIPK family, acting as decoys: modification of PTO by *P. syringae* effectors AvrPto and AvrPtoB triggers PRF-dependent ETI (Shan *et al.*, 2000; Abramovitch *et al.*, 2003; Lin and Martin, 2007; Rosebrock *et al.*, 2007; Mathieu *et al.*, 2014; Kraus *et al.*, 2016). Down-regulation of at least one tomato RPM1-INTERACTING PROTEIN 4 (RIN4) protein (SlRIN4-1) also seems to enhance PRF-dependent ETI (Luo *et al.*, 2009). In tomato plants, HopZ3_PsyB728a_ can suppress AvrPto-triggered immunity by acetylating key residues of the corresponding immune complex components, including host proteins PTO, SlRIPK, and SlRIN4, while also interacting with FEN (Jelenska *et al.*, 2021). However, it should be noticed that *P. syringae* strain B728a, which natively expresses HopZ3, is not a tomato pathogen (Chien *et al.*, 2013).

RIN4 also participates in the NLR RPS2 immune complex. *Pseudomonas syringae* AvrRpt2 functions as an ETI suppressor of AvrRpm1_Pma_-triggered RPM1-dependent defense response and enhances bacterial growth in susceptible plants lacking RPS2 (H. S. Kim *et al.*, 2005; M. G. Kim *et al*. 2005). However, in plant genotypes expressing RPS2, AvrRpt2 proteolytic cleavage of RIN4 triggers RPS2-dependent ETI (Axtell and Staskawicz, 2003; Mackey *et al*., 2003) (Fig. 3). *Pseudomonas syringae* T3E HopF2_PtoDC3000_ suppresses AvrRpt2-triggered RPS2-dependent immunity in Arabidopsis by preventing AvrRpt2-dependent cleavage of RIN4 (Wilton *et al.*, 2010), which HopF2_PtoDC3000_ ADP-ribosylates (Wang *et al.*, 2010) (Fig. 3). Interestingly, the same type of biochemical modification on the same protein (RIN4), likely in different residues, results in two completely different biological outputs since, as stated above, AvrRpm1_Pma_ ADP-ribosylation of RIN4 stimulates its phosphorylation, thus triggering RPM1-dependent ETI (Redditt *et al.*, 2019). XopAC_Xcc_, HopAR1_Pph_, and HopZ3_PsyB728a_ do not significantly suppress RPS2-dependent ETI (Feng *et al*., 2012; Lee *et al.*, 2015; Russell *et al.*, 2015). This is expected for XopAC_Xcc_ and HopAR1_Pph_, since RPS2 has not been shown to require their target RIPK, but is somehow unexpected for HopZ3_PsyB728a_ since this suppressor also targets RIN4 (Lee *et al.*, 2015).

## Some effector-triggered immunity suppressors target alternative RLCKs to also suppress PAMP-triggered immunity

As stated above, HopAR1_Pph_, HopZ3_PsyB728a_, and XopAC_Xcc_ all interfere with RLCKs such as Arabidopsis RIPK, or tomato PTO and FEN, to alter NLR-dependent immune signaling (Fig. 2). While these kinases participate in NLR signaling, RLCKs are also known to signal downstream PRR activation (DeFalco and Zipfel, 2021). The RLCK-VII family stands out among the several RCLK families for its role in immunity signaling (Liang and Zhou, 2018), and RLCK-VII members are targeted by several T3Es to suppress immune responses (Zhang *et al.*, 2010; Feng and Zhou, 2012). Amongst the different RLCK-VII isoforms, BOTRYTIS-INDUCED KINASE 1 (BIK1) (Veronese *et al.*, 2006) and AVRPPHB SUSCEPTIBLE 1 (PBS1)-like (PBL1) kinases are signaling hubs downstream of diverse PRR complexes, but many other RLCK-VII members participate in immune signaling (Rao *et al.*, 2018; DeFalco and Zipfel, 2021). RIPK also belongs to the RLCK-VII family, while PTO and FEN are yet to be grouped in a specific family, and all participate in ETI signaling (Liang and Zhou, 2018).

Interestingly, HopZ3_PsyB728a_ is also able to interact with PBS1 and BIK1, acetylating the latter (Lee *et al.*, 2015). This could explain HopZ3_PsyB728a_ activity as suppressor of flg22-mediated ROS production (Lewis *et al.*, 2014). Similarly, HopAR1_Pph_ targets BIK1, PBL1, and PBL2 to suppress PTI, by cleaving the same conserved motif shared by members of the RLCK-VII family, including RIPK (Zhang *et al.*, 2010; Russell *et al.*, 2015), while HopF2_PtoDC3000_ disrupts BIK1, PBL1, and PBS1 phosphorylation and suppresses PTI at the plasma membrane (Wu *et al.*, 2011; Zhou *et al.*, 2014). On its part, *Xanthomonas* XopAC_Xcc_ also modifies BIK1 and PBL1, but not PBS1, to suppress PTI (Feng *et al*., 2012). *Ralstonia* RipAC could also be interfering with PTI by indirectly affecting BIK1 homeostasis, through its targeting of a BIK1-regulatory ubiquitin ligase (Yu *et al*., 2022). Taken together, all these T3Es can suppress ETI and PTI by targeting different members of the same kinase family that participate in either defense pathway (Fig. 2).

## Mitogen activated protein kinase cascades as targets for both effector-triggered immunity and PAMP-triggered immunity suppression

MAPK cascades signal immunity downstream RLCKs in both the NLR- and PRR-dependent pathways (DeFalco and Zipfel, 2021), and are targeted by several ETI suppressors, which also suppress PTI. *Pseudomonas syringae* effector HopZ1a_Psy7B40_ suppresses PTI, and ETI triggered by the expression of effectors AvrRpt2, AvrRps4_Pph1448A_, and AvrRpm1_Pma_ (Macho *et al.*, 2010; Rufián *et al.*, 2015; Rufián *et al.*, 2021). HopZ1a_Psy7B40_ interacts with Arabidopsis MAP kinase kinase 7 (AtMKK7), acetylating a lysine residue required for full kinase activity and thus blocking MKK7-dependent immune signaling (Rufián *et al.*, 2021). By targeting MKK7, HopZ1a_Psy7B40_ can suppress PTI and ETI, and even systemic acquired defense (Rufián *et al.*, 2021) (Fig. 2). In the case of HopZ1a_Psy7B40_ and AtMKK7, interfering with a single target protein that participates in a common signaling step accounts for the suppression of different defense pathways.

*Pseudomonas syringae* effector HopF2_PtoDC3000_ interacts with Arabidopsis MKK5, ADP-ribosylating a key arginine residue and inactivating MKK5 function (Wang *et al.*, 2010). HopF2_PtoDC3000_ inactivation of MKK5 leads to suppression of PTI (Wang *et al.*, 2010), while HopF2_PtoDC3000_ action on RIN4 leads to the suppression of AvrRpt2-triggered RPS2-dependent ETI (Wilton *et al.*, 2010) (Fig. 2). In this case, it might seem that two independent targets, each contributing to a different defense pathway, account for independent suppression of PTI and ETI. However, RIN4 can be phosphorylated by MAP kinase 4 (MPK4) *in vitro* (Cui *et al.*, 2010), raising the non-mutually exclusive possibility that a MAPK module could be acting upstream of RIN4 to regulate plant immunity. Interestingly, ETI-suppressor HopZ3_PsyB728a_ also interacts with MPK4, but it does not seem to acetylate it (Lee *et al.*, 2015).

Other effectors suppress ETI by interfering indirectly with MAP kinase cascades. *Xanthomonas* effector XopQ_Xcv85-10_ inhibits ETI-associated cell death in resistant pepper and enhances bacterial growth in resistant pepper and tomato (Teper *et al.*, 2014). The T3E(s) triggering this ETI remain unidentified. XopQ_Xcv85-10_ interacts with tomato 14-3-3 isoform TFT4, which participates in ETI signaling, and this interaction is required for XopQ_Xcv85-10_-mediated virulence (Teper *et al.*, 2014). XopQ_Xcv85-10_ also interacts with SlTFT4 homologs from other plant species (Teper *et al.*, 2014). The eukaryotic family of 14-3-3 proteins participate in signal transduction by sensing the phosphorylation status of client proteins and modulating their activity, and members of this family are frequently targets for T3E interaction (Lozano-Durán and Robatzek, 2015). Interestingly, both the phosphorylation of Arabidopsis MAP kinase kinase kinases (MAPKKKs) and the ensuing activation of MAPK modules require certain isoforms of 14-3-3 proteins, which interact directly with several RLCKs and MAPKKKs, showing that 14-3-3 proteins act as scaffolds and activators of the RLCK-MAPKKK5 module (Dong *et al.*, 2023) (Fig. 2). In fact, several RLCKs have been identified that directly regulate MAPKKK activation downstream of PAMP perception (DeFalco and Zipfel, 2021). XopQ_Xcv85-10_’s interference with 14-3-3 isoforms to suppress ETI in pepper (Teper *et al.*, 2014) is intermingled with XopQ_Xoo_’s ability to suppress PTI in rice (Sinha *et al.*, 2013) but does not seem to be necessary for its detection by NLR Roq1 in *Nicotiana* (Adlung and Bonas, 2017; Schultink *et al.*, 2017). XopQ_Xcv_ belongs to the same T3E family as HopQ1_Pto_, also identified as an ETI suppressor (Martel *et al.*, 2022) that interacts with 14-3-3 proteins (Giska *et al.*, 2013; Li *et al.*, 2013) (Fig. 2).

## Suppression of the effector-triggered immunity triggered by effector-triggered immunity suppressors

After a first event of ETI suppression, the interplay between NLR-dependent defense and T3E-dependent suppression is a potentially recursive process, where a sequence of concatenated (or networked) ETI suppression events might take place. Such a network of epistatic interactions between several co-secreted effectors that result in cross suppression of ETI has been referred to as meta-effector interaction (Laflamme *et al*. 2020; Martel *et al*. 2022), a term originally coined for analogue interactions amongst T3Es comprising the secretome of the mammalian pathogen *Legionella pneumophila* (Kubori *et al*., 2010; Urbanus *et al*. 2016). Examples of such a complex iteration have already been outlined above.

In the very first description of ETI-suppressing effectors, AvrPphF_Pph1449B_ was shown to suppress the ETI triggered by unknown effectors secreted by its native *Pph* 1449B strain (Jackson *et al.*, 1999). But in bean cultivars carrying the appropriate plant resistance genes, AvrPphF_Pph1448B_ was itself detected, triggering ETI (Tsiamis *et al.*, 2000). This ETI was suppressed by effector AvrPphC_Pph1449B_ present in the same *Pph* 1449B native plasmid as AvrPphF_Pph1449B_, thus proving that an ETI suppressor can itself be ‘protected’ by another co-secreted effector in a natural pathosystem (Tsiamis *et al.*, 2000).

In a different experimental setting, *P. syringae* effector AvrRpt2 functions as an ETI suppressor of AvrRpm1_Pma_-triggered RPM1-dependent defense response, by cleaving RIN4 thanks to its cysteine protease activity (Coaker *et al.*, 2005; H. S. Kim *et al.*, 2005), while also suppressing PTI and enhancing bacterial growth in susceptible plants lacking the NLR RPS2 (M. G. Kim *et al.*, 2005). However, in plant genotypes expressing RPS2, AvrRpt2 cleavage of RIN4 triggers RPS2-dependent ETI, since RIN4 also participates in the RPS2 immune complex (Axtell and Staskawicz, 2003; Mackey *et al*., 2003) (Fig. 3). Thus, in RPM1 RPS2 plant genotypes, AvrRpt2 potentially functions simultaneously as an ETI suppressing and ETI-triggering T3E. The recursive process of ETI suppression continues here with another concatenated ETI suppression event, in this case with *P. syringae* T3E HopF2_PtoDC3000_, which suppresses AvrRpt2-triggered RPS2-dependent ETI in Arabidopsis by preventing AvrRpt2-dependent cleavage (Wilton *et al.*, 2010) (Fig. 3). HopF2_PtoDC3000_ exerts ADP-ribosylation on RIN4 (Wang *et al.*, 2010). While HopF2_PtoDC3000_ does not trigger ETI in Arabidopsis, another T3E of the same family (HopF2a from strain M302273PT) is detected by the NLR ZAR1, triggering ETI (Seto *et al.*, 2017), and thus further iterations of the ETI-suppression process might yet be identified.

In yet another example, *P. syringae* ETI suppressor HopZ3_PsyB728a_ can trigger ETI through unidentified NLRs in resistant genotypes of bean and tobacco (Vinatzer *et al.*, 2006). Several co-secreted T3Es, chiefly HopAF1_PsyB728a_, can in turn suppress HopZ3_PsyB728a_-triggered immunity (Rufián *et al.*, 2018a) (Fig. 4). Interestingly, HopAF1_PtoDC3000_ is yet another example of an ETI suppressor that can also suppress PTI (Washington *et al.*, 2016). The currently identified targets of HopAF1_PtoDC3000_, membrane-bound proteins METHYLTHIOADENOSINE NUCLEOSIDASE (MTN) 1 and 2, seem to exclusively participate in regulating ethylene production (Washington *et al.*, 2016), and thus it is likely that HopAF1 targets additional plant proteins to suppress ETI. HopAF1_PtoDC3000_ is membrane-bound by myristylation and palmitoylation (Washington *et al.*, 2016), a subcellular location fitting with HopZ3_PsyB728a_ and its targeted RPM1 immune complex (Lee *et al.*, 2015), suggesting the possibility that HopAF1 might associate with the complex.

In *Xanthomonas*, effector AvrBsT_Xcv75-3_ suppresses AvrBs1_Xcv85-10_-triggered ETI in resistant pepper plants (Szczesny *et al.*, 2010). AvrBsT_Xcv75-3_ belongs to the same effector superfamily as *P. syringae* HopZ1_Psy7B40_ and HopZ3_PsyB728a_ (Ma and Ma, 2016). AvrBsT interacts with pepper sucrose nonfermenting 1 (SNF1)-related kinase 1 (SnRK1), a putative regulator of sugar metabolism that is required for the induction of AvrBs1-specific HR (Szczesny *et al.*, 2010). SnRK1 does not interact directly with AvrBs1 but is presumably indirectly involved in the recognition of AvrBs1 by the corresponding resistance protein Bs1. Many other AvrBsT interactors have been described in Arabidopsis and pepper plants, but all but SnRK1 have been associated with AvrBsT-triggered ETI (Han and Hwang, 2017; Choi *et al.*, 2021). Interestingly, ectopic expression of XopB_Xcv85-10_ suppresses the immunity triggered by AvrBsT in pepper by *Xanthomonas* strain 75-3, naturally expressing AvrBsT (Schulze *et al.*, 2012). XopB_Xcv85-10_ expression in an AvrBsT knockout background provides no growth advantage to *Xanthomonas* 75-3 (Schulze *et al.*, 2012). XopB_Xcv85-10_ can also suppress PTI and interfere with the host vesicle trafficking. Interestingly, expression of an *xopB* mutant derivative defective in the suppression of ETI-related responses still interfered with vesicle trafficking and was only slightly affected on PTI suppression (Schulze *et al.*, 2012), suggesting that XopB_Xcv85-10_’s abilities to suppress PTI and ETI can be functionally separated. While the plant target(s) for XopB_Xcv85-10_ have not yet been identified, XopB belongs to the same effector family as HopD1_PtoDC3000_, which has been shown to target Arabidopsis transcription factor NTL9 (Block *et al.*, 2014).

## Effector-triggered immunity suppressors can also target co-secreted effectors

T3E targets have been traditionally searched for among plant proteins involved in immune signaling. In an interesting turn of events, Lee *et al*., (2015) demonstrated that ETI-suppressing HopZ3_PsyB728a_ interacted *in planta* with co-secreted T3Es AvrRpm1_Psy_ and AvrB3_Psy_, acetylating both at specific residues. This modification contributed to the suppression of RPM1-dependent ETI triggered by both AvrRpm1_Psy_ and AvrB3_Psy_ co-secreted T3Es (Lee *et al.*, 2015) (Fig. 4). HopZ3_PsyB728a_ also interacts with co-secreted T3E AvrPto1_PsyB728a_ (Lee *et al.*, 2015). Furthermore, in tomato plants, HopZ3_PsyB728a_ suppresses effector AvrPto1_PsyB728a_-triggered ETI not only by acetylating components of the immune complex (as mentioned above), but also by acetylating key residues of AvrPto1_PsyB728a_, which triggers immunity in the absence of HopZ3_PsyB728a_ (Jelenska *et al.*, 2021). Interestingly, AvrPto1_PsyB728a_ partially suppresses the HopZ3_PsyB728a_-triggered immunity when co-expressed in *N. benthamiana* (Rufián *et al.*, 2018a).

HopZ3_PsyB728a_ illustrates different levels on which a given T3E can suppress ETI, sometimes simultaneously, by altering (i) the virulence target (or decoy) to avoid its modification by the trigger T3E, as HopZ3_PsyB728a_-dependent RIN4 acetylation lowers its susceptibility to AvrRpm1_Psy_ or AvrB3_Psy_ modification; (ii) the defense partners of the target (or decoy) to interfere with the defense complex operation, as HopZ3_PsyB728a_-dependent acetylation of RIPK limits its ability to activate RIN4 by phosphorylation; and (iii) co-secreted T3E triggering ETI, as HopZ3_PsyB728a_ acetylates AvrRpm1_Psy_ and AvrB3_Psy_, likely to avoid their ETI-triggering action on RIN4 (Fig. 4). HopZ3_PsyB728a_ thus exemplifies how meta-effector activity can entail direct antagonistic interactions between ETI-suppressing and ETI-triggering effectors, or alternatively involve indirect interactions through shared host targets belonging to the same immune complex (Box 2).

Box 2. **Type III effector complexes with plant immune components and their subcellular localization**

HopZ3_PsyB728a_ is the only ETI-suppressing T3E shown to date to form multiprotein complexes with plant immune components and with co-secreted ETI-eliciting bacterial effectors (Lee *et al.*, 2015). However, our current views on ETI suppression may be an oversimplification, and such multiprotein complexes may be more common than expected. Indeed, it would be interesting to extend the search for complex interactions to other ETI-suppressing T3Es. For instance, HopAF1_PsyB728a_ might join the HopZ3_PsyB728a_ complex in *N. benthamiana* (Rufián *et al.*, 2018a); AvrRpm1, AvrRpt2, and HopF2 might be associated in the RIN4 complex (H. S. Kim *et al.*, 2005; Wilton *et al.*, 2010); *Xanthomonas* AvrBsT, AvrBs1, and XopB might be associated with SnRK1 and/or the corresponding NLRs (Szczesny *et al.*, 2010; Schulze *et al.*, 2012); and AvrPphC and AvrPphF might be associated with yet unknown plant targets (Jackson *et al.*, 1999; Tsiamis *et al.*, 2000). The composition of such complexes might vary depending on the plant host, as suggested by the interacting partners of HopZ3_PsyB728a_ in Arabidopsis apparently not including AvrPto1_PsyB728a_, although it does interact with it and modifies it in tomato plants, where HopZ3 suppresses AvrPto1_PsyB728a_-triggered immunity (Lee *et al.*, 2015; Jelenska *et al.*, 2021). In this regard, any given ETI suppressor might be considered as a potential partner for interaction and/or modification in a complex with its ETI-eliciting co-secreted T3E partner. Most of these T3E-defense protein assemblages are likely to associate to the plasma membrane, since this is the archetypal subcellular localization for plant immune complexes and since it has been estimated that over 30% of T3Es’ host targets are membrane proteins, reaching up to 50% for *P. syringae* T3E plant targets (Khan *et al.*, 2018). Many ETI suppressors are indeed associated to membranes via post-translational lipid modifications, like AvrRpt2, HopF2, HopAF1, HopAR1, or HopZ1a (Nimchuk *et al.*, 2000; Jin *et al.*, 2003; Robert-Seilaniantz *et al.*, 2006; Dowen *et al.*, 2009; Wu *et al.*, 2011; Lu *et al.*, 2013; Washington *et al.*, 2016). Further, the localization of a T3E suppressor might be influenced by its co-secreted effectors and plant targets. For instance, HopZ3_PsyB728a_ is mostly cytosolic when expressed alone but is stably recruited to the plasma membrane by its membrane-bound partner AvrB3_Psy_ (Lee *et al.*, 2015); HopQ1 localizes primarily to the cytoplasm but might undergo nucleo-cytoplasmic shuttling by association with its target 14-3-3 protein (Giska *et al.*, 2013); XopQ localization is dependent on phosphorylation of specific residues (Deb *et al.*, 2019); and HopAR1 and AvrRpt2 require *in planta* proteolytic processing to acquire their final subcellular localizations (Jin *et al.*, 2003; Dowen *et al.*, 2009; Lu *et al.*, 2013).

This latter mode of action raises an interesting question. HopZ3_PsyB728a_ acetylates residues in co-secreted AvrB3_Psy_ that are important for interaction with its plant target and for immune elicitation (Lee *et al.*, 2015). Whether these modifications only affect the modified effector ability to trigger ETI or its virulence function altogether has important evolutionary implications, since the latter case will imply that ETI suppression by a second co-secreted T3E entails the loss of the virulence function of the ‘protected’ effector. For other T3Es, amino acid residues essential for virulence on susceptible plants have been also described to be essential for ETI in resistant plants (Singer *et al.*, 2004; Ong and Innes, 2006; Wang *et al.*, 2010), but this might not always be the case (Gupta *et al.*, 2015), and thus this aspect of ETI suppression should be further investigated.

## Conclusion

### A desirable shift towards natural pathosystems and intra-secretome suppression

An effort should be made to analyse ETI-suppression events in the context of natural pathosystems, regardless of the technical difficulties involved, since the outcome of any plant–pathogen interaction will depend on the specific set of effectors of the pathogen and the genotype of the plant engaging in the interaction. Working exclusively in model plant systems would limit our understanding of the natural infection process. This should include analysing ETI suppression between T3Es encoded by the same strain, to avoid misleading results that sometimes come from heterologous experimental approaches. Further, when feasible the results obtained by co-expression of each functional pair of T3Es in the absence of co-secreted T3E of the same repertoire should be confirmed in the context of the full effector set of the corresponding bacterial strain.

However, development of novel experimental pathosystems can be challenging. Most significantly, for a pathosystem to be suitable for the analysis of ETI suppression, host-range determination is essential, as shown for the influential model defined by the different *P. syringae* pv. *phaseolicola* strains and the corresponding bean cultivars (Jackson *et al*., 1999; Tsiamis *et al*., 2000). This implies the availability of a variety of strains of the bacterial pathogen of interest displaying cultivar specificity on the corresponding plant host, and thus allowing the study of both compatible and incompatible host–pathogen interactions. Further, many technical hurdles hinder research on new experimental pathosystems, since both the bacterial pathogen and the plant host should be amenable to comprehensive genetic and molecular analysis. Hundreds of phytopathogenic bacteria whole-genome sequences are publicly available thanks to next-generation sequencing, from which virulence determinants can be identified through bioinformatic analyses. However, the functionality, interactions, and relative relevance during infection need to be experimentally validated. To this purpose the bacterial strain of interest should be easily cultured, amenable to transformation and/or conjugation, have a homologous recombination rate high enough as to allow the use of allelic exchange techniques, and characterized regarding its antibiotic resistance to facilitate selection. Basic molecular genetics tools and techniques should also be developed or else adapted from analogous model strains. Further requisites should be considered for the natural plant host, which in many cases may not have a fast life cycle or be problematic to grow using *in vitro* culture systems or growth chambers, or might not be amenable to transformation for heterologous gene expression and genetic analysis to allow the characterization of defense-associated genes. Bacterial infection assays on woody hosts are usually more technically challenging than infection assays on herbaceous plant hosts. Finally, establishing the proper set-up for the analysis of the interaction for a new pathosystem in the form of virulence or pathogenicity assays can be a complex and time-consuming task. Among other considerations, this implies selecting whether to perform the assays in whole plants or in excised organs, the mode (spraying, dipping, vacuum infiltration) and dose of bacterial inoculation, and the optimal timing for sample analysis. All these variables can potentially affect each other and the development of the infection with regard to bacterial multiplication, disease symptoms, or ETI-associated macroscopic cell death. The lifestyle of the pathogen, whether aerial or soil-borne, epiphytic, apoplastic, or vasculature colonizing, also heavily influences the efficiency of such interaction assays.

### A question of scale

The already described pairs of ETI elicitor/ETI suppressor T3E pairs should be further characterized to determine whether complexes comprising both ETI-triggering and ETI-suppressing T3Es and their plant host targets, decoys, and other defense components are involved in the process. It is becoming apparent that such complexes of bacterial virulence and plant defense components are likely to be a common occurrence, and the participant proteins cannot be characterized separately. Characterization of additional effector complexes could be a way to scale up the analysis of T3E interactions at the secretome level. While each effector complex could be considered as a minor hub of interacting T3Es, once several such clusters have been identified they might be linked together by shared effectors. While such an approach is unlikely to provide a whole secretome-level interaction network, it has the advantage of being based on validated experimental results fundamental to the physiological significance of plant–pathogen interaction. Further, experimentally characterized effector complexes in any given bacterial strain–plant cultivar pair can be used as a model to search, through bioinformatic means, for potentially similar nodes within the secretomes of the many already sequenced bacterial strains.

Such a bottom-up approach starting with the characterization of individual ETI-suppression events should be combined with top-down secretome-wide experimental approaches such as those recently performed with *P. syringae* (Laflamme *et al*., 2020; Martel *et al*., 2022; Ruiz-Bedoya *et al*., 2023) that highlight the complexity of intra-secretome interactions, which is key to the outcome of infection. Such methodology should be extended not only to other *P. syringae* strains but also to additional bacterial pathogen species like *Xanthomonas* or *Ralstonia*.

### ETI suppressors as potential host-range determinants

A lot of attention has been directed at how the host range of a given bacterial pathogenic strain is determined by the array of T3Es it expresses, regarding exclusively its potential detection by the NLR array present in the interacting plant genotype; that is, only the presence or absence of T3Es triggering ETI in the interacting plant has been traditionally considered. It is important to notice that the repertoire of ETI-suppressing T3Es present in a secretome can heavily influence the host range of a bacterial strain, by ‘cancelling-out’ the defense response triggered by co-secreted T3Es: a bacterial strain can evolve to avoid ETI by allelic variation, or loss of the detected T3E (and with it a potential beneficial function) but also by gaining a new, ETI-suppressing effector. Further, in interaction contexts where bacterial pathogens are close together, as for instance in the apoplast of the leaf during *P. syringae* infections, co-infecting bacterial variants can complement each other via the secreted T3Es (Rufián *et al.*, 2018b; Ruiz-Bedoya *et al.*, 2023). In this sense, T3Es can be considered as ‘common goods’ for the invading bacterial populations, and this concept can be applied to ETI suppressors.

### Most effector-triggered immunity suppressors also suppress PAMP-triggered immunity: multiple targets and/or PAMP-triggered immunity–effector-triggered immunity crosstalk

Many ETI-suppressing effectors are also capable of suppressing PTI. This raises three, non-mutually exclusive possibilities (Fig. 2). First, some T3Es suppress both ETI and PTI through their independent modification of multiple targets, with some targets participating in PRR-dependent signaling while others contribute to NLR-dependent signaling. This has been discussed above regarding several defense-suppressing effectors, such as HopZ3, HopAR1, or XopAC, that target multiple RLCKs of the same family, some involved in NLR-dependent responses, others involved in PRR-dependent defense signaling. In fact, the majority of T3Es have multiple targets, with an estimated 68% of T3Es targeting multiple proteins (Khan *et al.*, 2018). Further, T3Es frequently interfere with multiple members of a particular molecular category, with effectors affecting kinases, for example, targeting an average of 3.6 different kinases (Khan *et al.*, 2018). Secondly, some effectors such as HopZ1 or HopF2 target plant proteins that contribute to defense signal integration, and thus common to NLR- and PRR-dependent responses, like those configuring the MAPK modules. Finally, the interdependency between PRR- and NLR-dependent signaling pathways might account for cross-suppression in which the suppressing T3E alters either one of the defense signaling pathways. Such defense interplay is consistent with the existence of several characterized T3Es suppressing both PTI and ETI.

## Abbreviations:

ETI: effector-triggered immunity
NLR: NB-LRR or NOD-like receptor
PRR: pattern recognition receptors
PTI: PAMP-triggered immunity
RLCK: receptor-like cytoplasmic kinases
T3E: type III effector;
T3SS: type III secretion system
